# What Does Creativity Mean in Safety-Critical Environments?

**DOI:** 10.3389/fpsyg.2020.565884

**Published:** 2020-09-29

**Authors:** Samira Bourgeois-Bougrine

**Affiliations:** ^1^Université de Paris, LAPEA, Boulogne-Billancourt, France; ^2^LAPEA, Univ. Gustave Eiffel, IFSTTAR, Versailles, France

**Keywords:** creativity, safety, insight, improvisation, risk, unexpected, attention, neuropsychology

## Abstract

Safety in high-risk and time-pressured situations relies on people’s ability to generate new and appropriate solutions to solve unforeseen problems for which no procedures or rules are available. This type of ability is regularly associated with the concept of creativity. While psychology researchers have studied, for decades, how creative ideas and solutions are generated, this basic research has not made it into the more applied fields of human factors and neuroergonomics. Building on the research on the psychology and the neuropsychology of creativity, this paper will (1) address the question of what creativity means and what are its ties with problem solving and decision-making; (2) focus on the evidence of the creative processes, the underlying mechanisms, and the multiple psychological dimensions of the creative behavior involved in unexpected events in extreme environments such as Apollo 13 mission, United Airline Flight 232, and Mann Gulch wildfire; and (3) explore the implications for future research in the domains of neuroergonomics and differential psychology.

## Introduction

In a recent interview (Saraceno, 2018), James Lovell, commander of Apollo 13 mission, commented on the catchphrase “*Houston, we have had a problem*”^1^ saying, not without humor: “*the quote became iconic because it fits into millions of situations people experience every day. Every time you turn around, you seem to hear, ‘Houston, we have a problem.’ I wish I had copyrighted it!*” Apollo13 spaceflight crew and mission control team demonstrated considerable creativity and ingenuity under time and resource constraints. In this life-or-death situation, creativity was the critical pathway to survival. “It is a reactive force, triggered when all else fails, when the usual ways of doing things suddenly stop working and there is no choice but to discover or invent others” (King, 1997, p. 301). According to many experts, the ability to succeed under unexpected and extreme conditions involves “creative intelligence” (Orasanu and Fischer, 1997; Lagadec, 2009; Boy, 2013; Klein, 2013). This ability reflects the complementary roles and the integration of intelligence, which primarily focuses on finding the correct solution, and creative reasoning that allows the generation of new alternative and approaches (Jaarsveld et al., 2015).

Although references to creativity in safety are frequent, the underlying processes of the creative behavior in safety-critical environments received only limited attention. Building on several decades of research on the psychology and the neuropsychology of creativity, this article aims to advance our understanding of how experts create new solutions in extreme and unforeseen situations. These questions are not new but they will be addressed in this article from a new perspective. Traditionally, problem solving, judgment, and decision making in safety-critical environments have been discussed extensively in the literature based, particularly, on Rasmussen skill-rule-knowledge (SRK) model (for SRK; Rasmussen, 1985) and naturalistic decision-making framework (NDM; Klein, 2008). SRK model highlights three types of information processing according to the degree of conscious attentional control: (a) skill-based mode refers to the execution of highly practiced action in an automatic way, (b) rule-based mode consists of implementing prescribed rules with moderate control, and (c) knowledge-based mode involves high-level of conscious control and eight stages devoted to the analysis of the situation (activation, observation, identification, interpretation, or diagnosis) and to the formulation and the execution of an action plan (evaluating of the alternatives, task definition, procedure formulation, and execution). NDM relies on recognition-primed decision strategies, in which the familiarity with a situation is assessed and information is activated from memory. NDM research emphasizes the role of intuition in expert decision and “views intuition as an expression of experience as people build up patterns that enable them to rapidly size up situations and make rapid decisions without having to compare options” (Klein, 2015, p. 164). Building on SRK and NDM models, Orasanu and Fischer (1997) made an explicit link between creativity and aviation decision making (ADM) in critical situations. They considered that creating and implementing a solution when a problem is ill-defined or ambiguous is the most difficult action plan in ADM because it involves not only assessing the situation but also of creating a solution for a defined problem that has never been encountered.

Since the 1950s, psychology researchers have studied how creative ideas and solutions are generated. According to the 4Ps model, the psychology of creativity research covers four perspectives: Process (stages and the nature of the problem solving and decision making), Person (personality, intellect, temperament, experience, etc.), Product (e.g., when a thought becomes a course of action, a creative idea, procedure, physical object, etc.), and Press (impacts of factors in the physical and social environments). Moreover, the underlying mechanisms or brain function involved in the emergence of creative solutions have been investigated using the recent advance in neurophysiological [e.g., electroencephalography (EEG)] and neuroimaging [e.g., functional magnetic resonance imaging (fMRI)] studies of creativity. However, this basic research has not made it into the more applied fields of human factors and neuroergonomics.

Building on the 4Ps model (Process, Person, Product, and Press) and the neuropsychology of creativity, this paper will (1) address the question of what creativity means and what are its ties with problem solving and decision-making?; (2) focus on the evidence of the creative processes, the underlying mechanisms, and the multiple psychological dimensions of the creative behavior involved in unexpected and extreme events such as Apollo 13 mission, United Airline Flight 232, and Mann Gulch wildfire; and (3) explore the implications for future research in the domains of neuroergonomics and differential psychology.

## What is Creativity?

Creativity is the capacity to produce novel, original work that fits with task constraints and has value in its context. While intelligence relies on analytical thinking, the use of prior knowledge, and problem solving through the use of routine procedures, creative resolution of a problem involves the skill to make non-obvious connections in order to generate previously unknown solutions (Sternberg, 1997). Bruner (1983, p. 183 in Weick, 1993) described creativity as “figuring out how to use what you already know in order to go beyond what you currently think.” This description echoes the notion of potential, which refers to a latent state that may be put to use if a person has the opportunity. As part of an individual’s “human capital,” creative potential may remain latent and the individual may be aware of his/her potential or may be blind to it. Recent advances suggest that creative potential stems from numerous factors (cognition, personality, emotional, and environmental context); it can be defined, measured, and improved. It is based on 10 cognitive and conative dimensions (Lubart et al., 2013): Divergent thinking, Mental flexibility, Analytic thinking, Associative thinking, Selective combination, Openness, Tolerance of ambiguity, Intuitive thinking, Risk taking, and Motivation to create. These dimensions will be defined, in the Creativity Under the Gun: Evidence of Creativity in High Risk Environments section, when addressing the evidence of creativity in high-risk environments.

### What Are the Processes Involved in Creative Behavior?

The nature of the creative processes that produce original ideas that have value in their context could be considered within two conceptualizations (Fisher and Amabile, 2008): compositional and improvisational creativity. While in music and theater, there is an accepted distinction between composing and improvising, we will see throughout this article that in the context of safety, they are used interchangeably.

With regard to compositional creativity, one of the early sources of information is based on the introspection of eminent scientists such as Poincaré and Helmholtz. It allowed Wallas to formalize, in 1926, the four-stage iterative model of the creative process (Lubart, 2001; Bourgeois-Bougrine and Lubart, 2019): preparation (information gathering and preliminary analysis to define the problem), incubation (phase where there is no conscious work on the problem), illumination (when an interesting idea becomes conscious), and verification (evaluation, redefinition, and development of the idea; Iteration of the previous steps if the idea is unsatisfying). The initial stage could be considered as a “formless” situation where there is no structure, no task, and no problem to solve; ideas do not, indeed, present themselves as “problems capable of resolution or even sensible contemplation. They must be posed and formulated in fruitful and often radical ways if they are to be moved toward solution. The way the problem is posed is the way the dilemma will be resolved” (Getzels, 1979, p. 167).

Several stage models of creativity have emerged introducing changes and improvements to the four-stage model such as the creative problem solving (CPS) model. A recently accepted CPS model (Mumford and McIntosh, 2017) includes eight key sub-stages of creative thinking summarized in four components: understanding the challenge, generating ideas, solution planning and execution, and monitoring the results. This CPS model has similarities with *Knowledge based behavior* in SRK model (Rasmussen, 1985), which is frequently referred to in the safety context. Collaborative CPS in safety-critical events will be illustrated in the Creativity Under the Gun: Evidence of Creativity in High Risk Environments section.

Fisher and Amabile (2008) suggest that in compositional creativity, preparation can include the development of specific skills and obtaining the information needed to perform the task. In improvisation, such preparation cannot occur because immediate action is needed. Indeed, the main difference between improvisational and compositional creativity lies in the role of urgency. Time pressure is often what produces improvisation in the first place. Improvisation is considered as an unplanned, spontaneous, and intuition-guided action to achieve a goal; the actions contain both a high degree of novelty and a low temporal separation of problem presentation, idea generation, and idea execution (Vera and Crossan, 2005; Fisher and Amabile, 2008). Therefore, improvisation could be considered a deliberate creative process where a convergence between the “design and the execution of a new production takes place” (Miner et al., 2001, p. 314). There is no improvisation unless an action is taken (Weick, 1998). Individual or group improvisation systematically starts with a spontaneous and unplanned action when one does not have time to step back and think. As a consequence, improvisation in the safety context often refers to an outcome or a solution that emerges without planning.

Improvisation is often associated with the concept of bricolage, which involves a new combination of available resources (Adrot and Garreau, 2010). However, improvisation is different from bricolage because of the nature of the constraint in question: in the first case, the constraint is the lack of time that leads to “thinking in action” and, in the second case, the constraint is related to the lack of resources that leads to using the resources at hand. However, during improvisation, the pressure of time makes it unlikely to search for or obtain additional resources, increasing the likelihood of bricolage (Baker and Nelson, 2005; Adrot and Garreau, 2010).

Group improvisation involves a collective engagement in a joint creation. The creative performance is built through verbal and nonverbal interactions (e.g., speech, gestures, and movements). Musicians or theater actors perform as a group with no preparation, no previous experience of playing together, no script, and no director. They respond to each other as in a conversation, they are sensitive, attentive, and adapt to what other members of the group play or say. Miles Davis once said, “*Play what you hear, not what you know*.” Recent studies have identified changes in the brain during collective improvisation compared to individual or solo improvisation. Limb and colleagues (Limb and Braun, 2008; Donnay et al., 2014) used functional brain imaging to study the areas of the brain involved in musical improvisation. Professional, highly skilled jazz musicians played on a keyboard developed specifically for use in the context of brain imaging. During the collective improvisation (two musicians playing simultaneously during brain imaging), a strong activation of the dorsolateral prefrontal cortex (DLPFC) was observed due to the social context, which makes use of the working memory. In fact, in comparison with individual musical improvisation, the interaction between the two musicians requires paying attention to what is being played, thus placing a high demand on working memory. Interestingly, structured scoping study of improvisation in scientific literature (Frykmer et al., 2018) showed that collective improvisation in crisis management is a mere aggregation of individual improvisation at collective level, which is different from group improvisation in music and theater.

### Where Do Creative Ideas Come From?

The neuropsychology of creativity highlights possible underlying mechanisms that promote the emergence, the selection, and the implementation of creative ideas. The findings of domain-specific studies (e.g., creative writing, visual art making, melody improvisation, etc.) and psychometric tasks^2^ highlight the fact that creativity does not rise from a conceptual void but from an ongoing knowledge base development and personal past experiences (Madore et al., 2016; Abraham, 2017). Individuals accumulate a collection of knowledge and routines, which must be both readily accessible and flexibly organized to meet any situational demand. For instance, Bill Evans,^3^ one of the greatest jazz pianists of the second half of the 20th century, said that it took him 15 years of work from the time he first started improvising, at age 13, until he mastered the process of improvisation and was ready to create something truly valuable. Evans’s approach to music was a process of analysis followed by intuition. This highlights the pivotal role of intuition in improvisation and confirms the view of intuition as an expression of experience. However, as we will see in the next section, improvisation in safety-critical situations could involve insight problem solving instead of intuition. Insight is a sudden understanding on how to solve a problem while intuition corresponds to an association between a piece of information provided by the situation and information stored in memory (Klein, 2013).

Conceptual knowledge is represented within an extensive semantic network in the memory, with direct and strong connections between closely related concepts (e.g., Bees-Honey or Table-Chair). Although memory access and retrieval are critical to creativity, evidence suggests that it can also hamper original idea generation leading to cognitive fixedness (Beaty et al., 2017; Agnoli et al., 2020). For example, an excessive strength in semantic associations could lead to fixation on the strong associates and result in difficulties to transcend or to inhibit overlearned response, stereotypical associations, or salient concepts (Bendetowicz et al., 2018).

The ability to flexibly combine concepts stored in memory to form novel and useful associations requires the coactivation of large brain networks: the default mode network (DMN) and the control executive network (CEN). The DMN, which is hypothesized to be involved in spontaneous activation of concepts and experiences from memory, is known to support the divergent and open creative process; it is activated by diverse forms of tasks that require spontaneous activation of autobiographical and semantic memory, perspective-taking, and envisioning the future. However, the DMN is deactivated during attention-demanding externally oriented task (Buckner et al., 2008). The CEN is a goal-directed processing which (a) controls attentional shift from the external world to internal thoughts, (b) exerts a high cognitive control for the selection and integration of semantic concepts, (c) facilitates flexible switching between semantic categories during memory retrieval, and (d) mitigates sources of interference by suppressing salient conceptual knowledge. Using convergent and divergent tasks, Bendetowicz et al. (2018, p. 228) confirm that optimal creative performance “*requires controlled mechanisms such as strategic search and controlled retrieval in memory, the inhibition of interference caused by frequent and more salient associates, the integration or combination of the retrieved associates, and the selection and evaluation of a solution that satisfies the constraints of the task*.”

## Creativity Under the Gun: Evidence of Creativity in High Risk Environments

This section addresses traditional safety issues through the lens of the psychology and the neuropsychology of creativity which consider creative action within the CPS framework. Creative individuals such as artists, musicians, etc., who seek to express his or her feelings in an original way are considered to be involved in solving a problem. In safety-critical events, desperation drives individuals to create new solutions to survive. Three examples of successfully managed safety events where creativity was considered as one of the key factors will be tentatively analyzed from the perspective of individual and team’s creative behavior, and the neuropsychological underlying mechanisms of such behaviors highlighted. The first two case studies, Apollo 13 mission and United Airline Flight 232, are positive examples of collaborative problem solving and the third, Mann Gulch wildfire, is a successful example of individual insight problem solving. The analyzed data includes the following sources: official accidents reports, communications’ transcripts (cockpit voice recorder and technical air-to-ground voice communications), interviews, talks, and a testimony.

### Collaborative Creative Problem Solving in Safety-Critical Environments

#### Creativity in the Air: United Airline Flight 232

“Disaster in the air, are you ready?” was the subtitle given by the Alaska Air Safety Foundation to a talk given by Captain Alfred Haynes about Flight 232 of United Airline (UAL), one of the most celebrated cases of CPS (NASA-Dryden, 1991). The flight crew experienced severe difficulties controlling the airplane after a catastrophic loss of all hydraulic systems due to an explosion in the number two engine. The crew had been trained to manage “*one failure or double failures, but never a complete hydraulic failure*” (NASA-Dryden, 1991). The official accident report indicates that “*Douglas Aircraft Company, the FAA, and UAL considered the total loss of hydraulic-powered flight controls so remote as to negate any requirement for an appropriate procedure to counter such a situation…The simulator re-enactment of the events leading to the crash landing revealed that line flight crews could not be taught to control the airplane and land safely without hydraulic power available to operate the flight controls*” (National Transportation Safety Board, 1989). This begs the question: how did the crew deal with this complete unforeseen circumstance in the air with virtually no prior experience of flying an airplane under those conditions?

The flight crew engaged, during 45 min, in an efficient collaborative CPS demonstrating outstanding skills throughout the four-stage CPS process: understanding the challenge, generating ideas, solution planning and execution, and monitoring the results. During the whole event, the crew tried to make sense of what was going on by “reading into their situation patterns of significant meaning…Sensemaking is built out of vague questions, muddy answers, and negotiated agreements that attempt to reduce confusion” (Weick, 1993, p. 635). As Isaksen and Treffinger (1987) have argued the process started by a disorder phase (mess) during which the problem was defined. These authors distinguish between the discovery phase of the problem (something is wrong, unsatisfactory, or missing) and the preparation phase in which information is collected. The crew was aware that they had lost one engine. The captain called for engine failure checklist and noticed that something else was wrong as suggested in the following quote of the Captain of UAL-232 flight: “*the first thing it (the checklist) said was, close the throttle. And when I tried to pull the throttle back, it would not come back. Now, I’ve never shut an engine down in flight on a jet, so I did not know that when you pulled the throttle back, it did not come back. In the simulator, when you do it, it always came back. This one would not come back…*” The crew quickly understood that not only they had lost one engine but also the three hydraulic systems and had to deal with an additional problem (e.g., “phugoid”): “*we immediately determined that we could not control the airplane: it would not respond to the inputs of the crew… Besides losing all of our hydraulics, which gave us no control, we had a problem that I was not really familiar with, called ‘phugoid’…*” The two outboard engines were still running, but no flight controls were operative.

As in any ill-defined problem, the crew had a purpose (e.g., to keep the plane upright in the sky) but did not have a known means or obvious path to achieve it. While figuring out what is going on and trying to find an airport, they gathered information from air traffic control about possible landing areas (runways and highway), checked visually the external damage to the airplane, discussed the procedures, invited in the cockpit an off-duty DC-10 captain who volunteered his assistance, and contacted San Francisco area maintenance experts for help with the issue of the loss of hydraulics, etc.

This safety-critical event involved, indeed, numerous cycles of divergent and convergent thinking at each step of the process: the crew simultaneously generated and evaluated ideas and used these ideas to formulate implementation plans. The execution of these plans often led them to circle back as the output was inadequate until they figured out opportunistically a novel solution to operate the plane without any control. Every time the maintenance experts and the off-duty captain tried to find something that the crew could do, they had either already done it or could not do it, because of the loss of hydraulics. As the cockpit voice recorder (CVR) indicated, the crew collectively generated and tested possible solutions and courses of action in dealing with the loss of the hydraulic system, as well as the methods of attempting an emergency landing: “*if we had not let everybody put their input in, it’s a cinch we would not have made it…the way we flew the airplane (was): what do you want to do, I do not know, and let us try this, and you think that’ll work, beats me, and that’s about the way it went, really. If you read the CVR transcript, there’s a lot of that on there*.” This reflects a high degree of tolerance of ambiguity, openness, and risk taking which are among of the 10 dimensions of the creative potential (Lubart et al., 2013). Tolerance of ambiguity is characterized by the ability to solve, or at least to tolerate situations and/or information that are ambiguous, unclear, contradictory, or absent. Openness is the tendency to try out new things and to have new experiences; it is opposed to dogmatism and conformism. The idea of sensible or calculated risk taking is often associated with creativity (Bourgeois-Bougrine et al., 2020), and several researchers have argued for the need to measure risk taking in a variety of domains to better capture its complex nature (Sternberg and Lubart, 1995; Sternberg, 1997; Runco, 2015).

Moreover, the crew demonstrated a high-level of mental flexibility which is the ability to change points of view and to change initial cognitive frames in order to explore new directions, as suggested by the captain: “*we found that in order to stop a phugoid, you had to do the opposite of what you would normally do*.” This cognitive ability is synonymous to mental suppleness and to the ability to alternate between processing several kinds of information. They also stumbled upon solutions in an opportunistic way to several problems. For instance, with the help of the off-duty captain (who was assigned to the throttles), the crew managed to control the heading in synchronized effort: “*And we said (to the off-duty captain), give us a right bank, bring the wing up, that’s too much bank, try to stop the altitude, he’d try to respond. And after a few minutes of doing this, everything we’d do with the yoke, he could correspond with the throttles. So, it was a synchronized thing between the three of us, with (second first officer) still being able to do all his communications. So that’s how we operated the airplane, and that’s how we got it on the ground*.” This might represent an instance of rare and true group improvisation as experienced by musicians or theater actors “in the spirit of shared leadership, responsibility, mutual support, and care” (Nisula and Kianto, 2018, p. 485). Indeed, through verbal and nonverbal interactions, the crew coordinated and synchronized their actions in a spontaneous, unplanned, and never experienced way. Crewmembers were attentive and adapted to each other’s action.

Although the mood of the crewmembers was understandably negative (fear), there were no apparent symptoms of panic as suggested by the CVR and the Captain in his talk: “*although we did not appear to be panicked…an airplane about to roll onto its back at 35,000' is pretty scary, so you just do anything you can to make it stop*.” Provided that they are not associated with extremely high-level arousal, negative mood states might increase the capacity to consider multiple alternatives because of enhanced persistence (Nijstad et al., 2010).

#### Distributed Creative Teams: Apollo 13 Mission

During the commemoration of 45th anniversary of Apollo 13 mission, Jim Lovell^4^ said “*The flight was a failure in its initial mission. However, it was a tremendous success in the ability of people to get together, like the mission control team working with what they had and working with the flight crew to turn what was almost a certain catastrophe into a successful recovery*.” Similar to the abovementioned case (UAL-232 flight), the teams demonstrated outstanding collaborative CPS skills, high degree of divergent thinking, tolerance of ambiguity, openness, risk taking, and mental flexibility. However, in the case of Apollo 13, the teams were distributed between space^5^ and the ground^6^ and the creative effort lasted about 80 h after the blast. The transcript of the technical air-to-ground (TAG) voice communications^7^ shows that through constant communication, trust, and care, the flight crew and mission control established and maintained a shared understanding. They monitored and evaluated the results of their actions, provided feedback, and adapted plans.

The blast that occurred 200,000 miles from earth at 55 h:55 min into the mission led to a major loss of power, oxygen, heating, disturbed the supply of water, and forced the crew to abandon the command module (CM) and use the lunar module (LM) as a lifeboat. Immediately after the blast, the creative process started with a phase of mess-finding in which the problem was defined. Both teams engaged simultaneously in troubleshooting the possible issues to make a sense of the erratic readings as suggested below in the followings TAG transcript:

- “*055:55:51 Liebergot: Okay, flight, we have got some instrumentation funnies. Let me add them up*. (In Mission Control Center in Houston, the flight controllers monitor the ship’s remote telemetry)
- *055:55:58 Lousma: Okay, stand by, 13. We’re looking at it. [Pause.]*
- *056:03:17 Swigert: Okay, Houston. Are you still reading 13?*
- *056:03:20 Lousma: That’s affirmative. We’re reading you. We’re trying to come up with some good ideas here for you*.
- *056:03:29 Haise: Okay. Let me give you some readings…”*

As the teams examined the gauges, they started gaining a greater insight into the magnitude of the failure ahead of them. They stayed calm and engaged in numerous cycles of divergent and convergent thinking. For instance, they brainstormed ideas after Jim Lovell’s announcement of something leaking from the ship:

- *“056:09:07 Lovell: We are venting something out into space…It’s a gas of some sort*.
- *056:09:29 Kranz: Rog. (Pause) Okay, let us everybody think of the kind of things we might be venting…”*

Once the lunar landing was aborted, the big questions were: how do we return to earth safely? How to deploy the capability of the LM? How will we overcome the damaged alignment system? etc. After a day and a half in the LM, a warning light showed that the carbon dioxide had built up to a dangerous level. But the CM’s square-shaped canisters, which remove carbon dioxide from the spacecraft, were not compatible with the round openings in the LM environmental system. Mission control devised and transmitted to the fligthcrew a way to attach the CM canisters to the LM system by using plastic bags and cardboard and to tape all materials carried on board. This outcome is an example of creative bricolage under resources constraints.

Moreover, the teams came up with five return-to-earth options and developed an alternative procedure to use the Sun as an alignment star as a result of the damages caused by the explosion to the alignment system (Granath, 2015). Among others, the teams generated and discussed ideas to solve the problem of the entry procedure:

- “110:23:19 Lousma: Jim, we have had a lot of people working on the entry procedures, and they will be continuing to do so. We got a few ideas we would like to toss at you so you can start thinking about them…?”

Similar to members of creative innovation networks (Gloor, 2006), the flight crew and mission control team’s work was interdependent, based on trust, respect, reciprocity, and consistency. All along, the knowledge was questioned, the problems redefined, and the solutions generated through an iterative process of CPS. This process involved prototyping solutions with what the crew had on-board and testing new procedures in the simulator. When asked if the question of survival ever came up, Jim Lovell showed outstanding emotional control: “*Honestly, no, we never had that thought. As long as the situation wasn’t hopeless, we thought positive*” (Saraceno, 2018).

### Problem Solving Through Insight

#### Creative Desperation: Mann Gulch Fire

On August 1949, Wagner Dodge and his 15 crewmembers were running uphill for safety, when he realized that the fire was only 50 yd. away behind them, and they could not outrun it. He stopped to light an escape fire as his testimony indicates^8^: “*the fire was too close, in my estimation, to continue farther. At this point, I stopped the crew and explained to those nearest me (at least eight men) that we would have to burn off a section of the light fuel and get into the inside in order to make it through… After setting a clump of bunch grass on fire, I had an area of 100 feet square that was ablaze…for all my hollering, I could not direct anyone into the burned area…within seconds after the last man had passed, the main fire hit the area that I was in…This lasted approximately 5 min, and I was able to sit up within the burned area…*” Dodge’s intention was to provide the crew a burned over, fuel-free zone but none of the crew followed his order to dive in the ashes that saved his life. Lillquist (2006, p. 567) reports that “*when later asked by the Board of Review whether he had been taught to set an escape fire in such a situation, Dodge replied Not that I know of. It just seemed the logical thing to do. I had been instructed if possible to get into a burned area*.” As the burned area was behind the “wall of fire” that was about to engulf him, he created the escape fire to “get into a burned area” of his making.

In his analysis of this disaster, Weick (1993, p. 642) described the escape fire as a “burst of improvisation” in the face of an inconceivable life-threatening event. This improvisation does not rely on intuition but on insightful problem solving (Klein, 2013). Insight or “Aha Moment” requires a spontaneous and sudden reorganization of the elements of the problem, a perspective shift to find the correct solution, and a transition from one mental model to another that is more satisfying and bringing suggestions for new actions that can remedy the tensions inherent in the previous mental model (Klein, 2013; Abraham, 2018). Klein and Jarosz (2011) referred to it as a “creative desperation path” triggered in a situation of imminent danger when the individual is confronted with an impasse resulting from deliberate and often desperate efforts to escape it. In contrast to Wallas’s four-stage model, when a person reaches an impasse and needs a quick breakthrough, a sudden reframing or restructuration of their mental model of the situation may occur without any deliberate preparation or incubation. Insight or revelation requires a high degree of mental flexibility to generate a new interpretation of the problem and to restructure it.

Referring to the escape fire, Weick (1993, p. 638) indicates that “what we do not expect under life-threatening pressure is creativity.” This leads us to two questions: why did his crew members not see escape fire as a lifesaving solution? and why was Wagner Dodge the only one who came up with this solution? The first question has received an extensive analysis from several perspectives. For instance, Weick (1993) suggested that Dodge’s failure to get his crewmembers in the escape fire, that resulted in the death of 13 men, was due to the “collapse of sensemaking” and the disintegration of role structure in this minimal temporary organization. While the second question remains difficult to answer, we will provide in the next section some basic research evidence of the underlying mechanisms of insight problem solving compared to traditional analytical approaches (e.g., conscious and deliberate search through a space of potential solutions).

#### The Underlying Mechanisms of Insight Problem Solving

Using psychometric tasks, EEG recording, brain imaging, and eye tracking, recent laboratory studies (Kounios and Beeman, 2014; Salvi et al., 2015) have attempted to answer two challenges: identifying the differences in cognitive and neural mechanisms in insight vs. analytical responses and the existence of an unconscious process preceding a conscious response. The results indicate differences in brain activation and eye movements of participants depending on the type of problem solving. Successful problem solving through insight involves a transient reorientation of attention inward while preparing and solving a problem. Kounios and Beeman (2014) showed that solutions that emerge from insight, compared to analytical solutions, were associated with an intense activity of gamma waves (40 Hertz) preceded by a burst of alpha waves (about 10 Hertz). The increase in gamma activity is considered to be the main correlate of the insight experience: it allows the link between treatment in different areas of the brain to build a coherent percept (Tallon-Baudry et al., 2005), and it occurs when the participant finds the solution to the problem, in a brain region involved in semantic integration (St George et al., 1999). The burst of alpha, on the other hand, indicates that the brain limits the flow of external visual information in order to avoid distraction, which could disturb the emergence of the solution by insight. The authors point out that in normal circumstances, when asked a difficult question, we often tend to look away from the person who asked that question or even briefly close our eyes during the search for an answer. As the participants in this experiment were instructed to look at the center of the screen, the increase in alpha waves is a compensatory phenomenon of the brain, which directs attention inward in order to protect the emergence of the solution. In other words, reducing temporarily interfering visual inputs allows the solution to pop into awareness. The results observed by exploring brain activity were confirmed by a study that used eye tracking technique to study attention in a similar experimental design (Salvi et al., 2015). The changes in the duration and frequency of blinking and eye fixation are overt indicators of the modulation of attention. Immediately prior to solutions, participants blinked longer and looked away from the problem more often when solving it by insight than when solving analytically. Spontaneous eye blinks are hypothesized to be actively involved in the release of attention from external stimuli to internal thoughts and tend to occur at breakpoints of attention, such as the end of a sentence while reading, a pause by the speaker while listening to a speech, etc. A recent study (Nakano et al., 2013) suggested that eye blinks are actively involved in the process of attentional disengagement during a cognitive task. The control of attentional process facilitates the shift of attention between external task and internal thoughts, the inhibition of most common response, the access and the combination of remote conceptual knowledge.

To sum up, insight problem solving involves a shift of attention inward and a “transient sensory gating” (Kounios and Beeman, 2014, p. 80). Despite the limitations of the laboratory approach, the neuropsychological studies of creativity open up new avenues for future research to understand the cognitive process and the underlying mechanisms of creative and insight problem solving in life critical-situation.

## Concluding Thoughts: Implication for Future Research

Drawing on the aforementioned literature and safety events, we propose a definition of creativity in safety-critical environments as “the capacity of expert individuals and teams to create original, unusual, and adapted solutions to solve unforeseen problems in life-critical situations, for which there is no prescribed procedure or obvious solution to apply.” Contingent on this definition, the solutions must contain both a high degree of novelty and adaptability, which also strike others as being interesting or clever (Kellner and Benedek, 2017). The solutions or “products” should be distinguished from the process that leads to the emergence of the successful outcome. In contrast to the existing literature on experts’ decision-making, insights from the psychology of creativity research suggest that creative behavior involves not only intuition as mentioned in the NDM model but also the combination of several cognitive and conative factors such as divergent thinking, mental flexibility, tolerance of ambiguity, etc.

Among the many implications for future research, two main issues could be addressed. The first issue, which represents a new research opportunity for neuroergonomics, would explore the neural basis of creative and insight problem solving in life critical-situation. The second issue falls within the traditional boundaries of differential psychology and would address the nature of the attributes of creative individuals in safety contexts. The ultimate goal is to improve operational training and required skills to deal with the unexpected and to explore design principles for human-machine systems that would support creative behavior whenever required.

### What Would Be the Underlying Mechanisms of Creative and Insight Problem Solving in Life Critical-Situation?

In life or death situations, acute stress and anxiety can lead to severe performance impairment due to cognitive fixation and mental block (Jouniaux, 2001). Attention and cognitive tunneling on specific symbology or stimuli could result in failure to detect potentially critical events that do not fall within the attended region (Jarmasz et al., 2005). Based on the neuropsychology of creativity, we can hypothese that these stress reactions would potentially limit the shift of attention between external task and internal thoughts, prevent the “sensory gating,” and hinder the inhibition of most common response as well as the access and the combination of remote conceptual knowledge.

Therefore, a particular attention should be devoted to the study of the underlying brain function involved in creative and insight problem solving related to operational performance in a simulator or virtual reality environment. This would require identifying and analyzing operational safety-critical events where there is evidence of creativity in line with the aforementioned definition of creativity in safety-critical environments. A simulation of the selected events will provide the opportunity for neuroergonomics researchers to explore in objective way (e.g., using eye tracking and brain imaging technics) the following issues: (1) the mental processes and environmental cues that lead to or prevent the emergence of new ideas and solutions in life critical events, (2) ways to optimize attention control and emotional regulation when solving operational problems under extreme stress such as mindfulness training (Meland et al., 2015; Abraham et al., 2019), and (3) the design principles for human-machine systems that will optimize the attention span and focus to avoid “locking” the user on an unsuccessful path in solving unexpected and extreme problems (Klein, 2013).

### What Would Be the Attributes of Creative Individuals in Safety Context?

Experts agree that “*If the unforeseen were the norm, crew training should be fundamentally oriented to deal with it - particularly stimulating pilots’ capacities for judgment and creativity, essential qualities for this purpose*” (AAE-Académie de l’air et de l’espace, 2013, p. 39). For instance, reflecting on what defined the first astronauts, Jim Lovell said: “*Originally, we were all test pilots. We sort of lived on the edge. We tested unproven airplanes for the military; we always expected something to go wrong*” (Saraceno, 2018). Moreover, Alfred Haynes served as a pilot in the Navy during the Korean War for 4 years before joining United Airlines. This confirm the role of experiences in the development of knowledge and highlight the need to (1) understand how past experiences in difficult conditions shape the creative potential to instigate CPS and decision making in stressful and extreme situations and (2) explore the nature of trainings that would enhance the creative potential of ordinary frontline operational without having to live on the edge.

To be able to develop these trainings, there is a need to identify and measure the required abilities and skills. As explored in the previous section, the creative process in life critical events is oriented toward damage control and reflects the creative potential of experts. To make a sense of the unfolding events and to come up with appropriate and unusual solutions, several cognitive and conative factors are critical such as divergent thinking, mental flexibility, tolerance of ambiguity, analytical skills, etc. As it has been suggested (Lubart et al., 2013), the assessment of the creative potential profile would help to (a) identify the strengths and weaknesses of each person in relation to the average profile of his group or to the top performers in his domain and (b) to tailor training that target weaknesses in specific dimensions. A multidimensional approach has been adopted to detect of the creative potential in children, adolescent, and adults such as managers or designers (Caroff et al., 2018). Similar approach could be used to determine (1) whether there is a particular profile of the creative potential or skills that facilitate insight and CPS in life critical-situation and (2) how these skills and abilities could be developed?

In conclusion, we would like to emphasize that the successful outcomes in safety-critical situations rely on four sources of resilience: creativity, role system, attitude of wisdom, and respectful interaction (Weick, 1993, p. 638). To the question “Disaster in the air, are you ready?,” Captain Haynes answered “No, you are never ready. But you might be prepared.” We hope that revisiting the role of creativity in safety opens up multiple implications for future research that would contribute to the reinforcement of resilience.

## Author Contributions

SB-B prepared and wrote the manuscript. It is based on a review and synthesis of knowledge from multiple disciplines and sources including her own research and experience.

### Conflict of Interest

The author declares that the research was conducted in the absence of any commercial or financial relationships that could be construed as a potential conflict of interest.

## Footnotes

^1^The legendary line delivered by Lovell is “Houston, we have had a problem” and not the familiar “Houston, we have a problem” made especially popular by the Tom Hanks movie. https://history.nasa.gov/afj/ap13fj/08day3-problem.html

^2^Psychometric tasks: alternative uses task (AUT) and remote associates test (RAT). The AUT assess divergent thinking and the RAT measures the ability to see relationships between things that are remotely associated.

^3^http://www.openculture.com/2012/04/the_universal_mind_of_bill_evans_advice_on_learning_to_play_jazz.html

^4^https://www.nasa.gov/content/members-of-apollo-13-team-reflect-on-nasas-finest-hour

^5^Flight crew – Commander: Jim Lovell; Command module pilot: John Swigert; Lunar module pilot: Fred Haise.

^6^Mission control (Houston): flight directors, capsule communicators, engineers…

^7^https://history.nasa.gov/afj/ap13fj/21day5-batterycharge.html

^8^Testimony of smokejumper foreman Dodge. https://www.nifc.gov/safety/mann_gulch/event_timeline/event3/documents/Pages%20117_118%20from%20Board_of_Review_%20Sept%2026_28_1949.pdf
